# 

**Published:** 1979

**Authors:** 


JULY/OCTOBER 1979

VOLUME 94 (iii/iv)

NUMBER 351/352

Published Quarterly

Annual Subscription £6 Post Free

### BRISTOL MEDICO-CHIRURGICAL JOURNAL

### Contents

3 Postgraduate Medical Education in the South West

J. E. Cates

13 Obituary
Dr. R. I. S. Gordon, M.D., F.R.C.P., F.R.C.R.

15 Abstracts

Established 1883

### BRISTOL MEDICO-CHIRURGICAL JOURNAL

Incorporating the Medical Journal of the South West

### Editorial Committee

EDITOR
*Dr. D. S. Reeves*
*Southmead Hospital, Bristol, BS10 5NB*
ASSISTANT EDITOR
*Dr. A. P. Radford*
MEMBERS
*Dr. Rhys Davies*
*Dr. M. B. Lennard*
*Dr. D. W. Wright*
*The Hon. Treasurer of the Society*
*The Hon. Secretary of the Society*
SECRETARY
*Mr. B. P. Jones, The Library, Medical School,*
*University Walk, Bristol, BS8 1TD*

### Bristol Medico-Chirurgical Society

PRESIDENT 1979/80
*Dr. D. S. Ractliffe*
PRESIDENT ELECT
*Dr. N. J. Brown*
HONORARY SECRETARY
*A. J. Webb, Esq., 7 Percival Road, Bristol, BS8 3LE*
HONORARY TREASURER
*Dr. J. Penry, Southmead Hospital, Bristol, BS10 5NB*

The Society was founded in 1874. In 1883 publication of the Journal began and has continued quarterly ever since then. During the years 1949 to 1962 it appeared under the title of 'The Medical Journal of the South West'.

### Notice to Contributors

*The Journal is especially concerned to provide a place for the recording of medical thought, interests, and practice in and around Bristol. It therefore welcomes articles that, as well as being of immediate interest to members, will document the local and contemporary medical scene for our successors.*

*Original articles are invited provided that they have not been published elsewhere. Illustrations should be supplied ready for reproduction. Line drawings should be in Indian ink on firm white paper or board. The author will be informed if it is found necessary to ask him to pay for the printing of photographs etc.*

*The following contributions will be especially welcome; notes of forthcoming events, meetings held, degrees conferred, staff appointments, honours and public appointments and other local news.*

*Advice on preparation can be obtained from the Editor.*

Printed by the University of Bristol Printing Unit

### University of Bristol

### FACULTY OF MEDICINE

### The Degree of Doctor Philosophy
### June 1979

Christopher John GASKELL

### October 1979

Clive Beverley Goldsworthy JENKINS
Bijan RADMEHR
Robert SKIDMORE

### The Degree of Doctor of Medicine
### June 1979

Michael Henry CULLEN
Alan Raymond HEARN

### October 1979

Christopher Michael ASPLIN
Clive John Charlton ROBERTS

### The Degree of Master of Science in Medical Sciences
### October 1979

Nelson Amaitari BEREPUBO

### Final Examination for the Degree of B.V.Sc.
### June 1979

**WITH HONOURS**

Marion Janet MAGGS
with Credit in Veterinary Medicine
Graham Allen MUNROE
Timothy John PARKINSON
with Credit in Veterinary Medicine
Cyril RUTLEDGE
Susan Anne WRIGHT
with Distinction in Veterinary Medicine

**PASS**

Michael BAILEY
Jennifer Helen Anne BOWEN-DAVIES
David John BREWER
Sarah Grace CRIMP
Robert Harold DAVIES
Robert Anthony DONE
Fiona Mary HOWARD
with Credit in Veterinary Surgery
Alan Leslie HUGHES
Isobel Margaret KINNIER WILSON
Zoe Jane MALDEN
Judith Sarah MARTIN
Colin Patrick MELBOURNE
Christopher Anthony William MITCHLEY
William John PEPPER
Robert Graham PETTITT
Stephen William POINTING
Deidre Elizabeth Anne SQUIRE
Julie THURGOOD
Graham Ernest WHEELER
Sally Anne WILLIAMS
Gillian Elizabeth WOOLLEY

### September 1979

**PASS**

Peter Bonham Selwyn BAZELEY
Wilfred Timothy Fine HOSKEN
Carol Isabel YOUNG

### Final Examination for the Degrees of M.B., Ch.B.
### June 1979

**WITH HONOURS**

Gabrielle Helen KINGSLEY
with Distinction in Community Health
Paul Gerard NEWRICK
with Distinction in Surgery
Hardev Singh PALL
with Distinction in Child Health
Maureen REYNOLDS
with Distinction in Obstetrics and Gynaecology and in Community Health
Joyce Isabel WILSON
with Distinction in Mental Health

**PASS**

Kathryn Robina AGASCAR
Michael John ALLEN
John Barry ANDERSON
Peter John BENTON
Teresa Jane BLACK
David Francis BOOTH

Julian Henry BRADLEY
Amanda Margaret Meredith BROWN
Anthony Frank Treacher BROWN
Grahame John BROWN
Colin Charles BURGESS
Simon James BURRELL
Catherine Marian CORBISHLEY
Barbara Elizabeth CRESSWELL
Jane Evelyn CRESSWELL
Jane Wallace Ainsworth CURLEY
David Allan Brett DANCE
Nigel William DAVEY
Anthony Geraint DAVIES
David Michael DAVIES
Caroline Mary DENCER
Jonathan Peter Crispin DE PASS
Richard William DRAYSON
Eric George DUNCAN
Barbara Helen DUNNING
Nigel Lyons Gwynne EASTMAN
with Distinction in Community Health
David James EDDYSHAW
Caroline Hannah Dorothy FALL
William Jeremy FERGUSON
David Charles FISH
James Loman FITZGERALD
Annable Brigid Mary FOOT
Anna GAJEK
Keith GILLESPIE
James Martin GLEDHILL
Michael Stewart GOODALL
David Michael GREET
Deborah HADDON-CAVE
Margaret Catherine Helen HARKNESS
Corinne Elise HARRIS
Martin David HARRISON
William Joseph HAWKEN
Jeremy HAYNES
James David HILL
Rosemary Elizabeth HILL
Adrian John HOBBS
Mark Peter HOLLIDAY
Anne Sheila HOLLMAN
Martyn Wayne HUGHES
Alison Mary HUMPHREYS
Adrian Mark JENNINGS
Bharat Kumar JINDAL
Lindsay Carol JONES
Lynne Myfanwy JONES
Janet Elderton KELSALL
Terence KIRKPATRICK
with Distinction in Child Health
David Paul KNIGHT
Paul John LAKING
with Distinction in Mental Health
Edward John LARCOMBE
Bruce Baird LETHAM
Leun Ting LEUNG CHEE HANG
Thomas Holt LLOYD
Robert Frederick LOGAN
Ian Robert LONGHORN
Margaret Fiona LOTT
Malcolm LYONS
Charles Francis MACADAM
Roderick Ian MACKICHAN
Judith Douglas MALONE
John Charles MARKS
Philip John MATTHEWMAN
Graham Robert Burton McGEOCH
Aileen Joan MILLER
Alan MORDUE
Greta Leigh MUSHET
Howard Christopher NAYLOR
Julia Sibyl NELKI
Martin Charles NEWMAN
Helen Diana NICHOLSON
Diane Elizabeth NURSE
Kerry Michael O'CONNOR
Jane Louise OWEN-JONES
Vivien Mary PARKER
Nicholas David Thomas PIMM
Anton Louis POZNIAK
Christopher Patrick Joseph PRICE
William Thomas McLean RANSOM
Annette Mary RIISNAES
Jane Suzanna ROACH
Anthony Philip ROBERTS
Glenn Anthony ROBERTS
Karen Anne ROBINSON
Nicholas ROONEY
Peter Andrew ROWE
Virginia Helen ROYSTON
Christopher John SALISBURY
Julie Doreen SHERLOCK
Stanley George SHEPHERD
Michael David SHIELDS
David Barry SHORTLAND
Nigel James SPARROW
Clare Karola STRONG
Jennifer Elizabeth STUTLEY
Ashwani Kumar SURI
Paul Rhodri THOMAS
Alastair Grant THOMSON
Alison Mary TODD

Elizabeth Jane TODD
Leslie John TOOP
Ruth Marjorie TREBBLE
Jean Elizabeth TYLER
Ewan Glyndwr WALTERS
Martin Peter WARD PLATT
George Philip WHITE
Elizabeth Ruth WHITTLE
Guy Stephen WILDY
Dorothy Jane WILLIAMS
Gerard Trevor WILLIAMS
with Distinction in Surgery
Stephen WILLIAMS
Simon David WORSLEY
Judith Irene WYATT
Philip Andrew YATES

**December 1979**

**PASS**
Jeremy David CORSON
Sara Ann EAMES
Timothy Guy PEACOCK
David Charles SIMPSON
Caroline Yelverton WARREN-BROWNE

**Final Examination for the Degree of B.D.S. (Section II) December 1979**

**WITH HONOURS**
Andrea Maxine FINDLAY
Newton Daniel JOHNSON
Pamela Anne NORMAN
Rachel Ann PINDER
Andrew Michael PROSSER
Sian ROBERTS

**PASS**
Khairiyah ABDUL MUTTALIB
Alan Paul BARCLAY
Duncan BARCLAY
Barbara Yvonne BEVAN
Sheila Anne BIRKS
William Knights BRADY
Lynne Margaret BULLOCK
Simon Alan CARLYLE
Helena Gizela Victoria CEMBROWICZ
Trevor Charles CHAPMAN
Jennifer Elizabeth Ann CHIN
Brian James DAVIDSON
Stuart Charles DAVIS
Michael John FARDY
Roderick Allan MILNE
Paul Stuart MITCHELL
Christopher David Nicholas MORRIS
Deirdre Kathleen MULROONEY
Lesley Ann NOLAN
Philip Brian NOWELL
Nawshrat Sadrudin RAHIM
Stephen John ROBEY
Richard ROBINS
Alison Jane ROE
Paula Jane TROWER
Paul William WATSON
Joanna Margaret WHITTARD
Charles Richard WILBY
Hilary Jacqueline WILLIAMS
Claire Susan WOODS
Wei Luen YAP

